# Rates of Spontaneous Abortion in Israel Before and During the COVID-19 Pandemic

**DOI:** 10.1001/jamanetworkopen.2023.0233

**Published:** 2023-02-21

**Authors:** Yael Travis-Lumer, Yair Goldberg, Arad Kodesh, Abraham Reichenberg, Sven Sandin, Sophia Frangou, Stephen Z. Levine

**Affiliations:** 1 Faculty of Data and Decision Sciences, Israel Institute of Technology, Haifa, Israel; 2 Mental Health Department, Meuhedet Health Services, Tel Aviv, Israel; 3 Department of Psychiatry, Icahn School of Medicine at Mount Sinai, New York, New York; 4 Department of Psychiatry, University of British Columbia, Vancouver, British Columbia, Canada; 5 School of Public Health, University of Haifa, Haifa, Israel

## Abstract

This cross-sectional study uses electronic health record data to compare monthly incidence rates of spontaneous abortion in Israel before and during the COVID-19 pandemic.

## Introduction

Spontaneous abortion (SA) (pregnancy loss before 20 weeks’ gestation) affects between 11% and 20% of pregnancies.^1^ Studies of the SA rate during the COVID-19 pandemic focus mainly on vaccine safety.^2^ However, exposure to the prolonged biopsychosocial adversities of the pandemic may have cumulated in a perfect storm of risk factors (eg, infection and stress during pregnancy)^3,4^ associated with an increased SA rate. Alternatively, as found for other pregnancy-related outcomes, COVID-19 attenuation strategies may have been associated with reduced stress and, hence, SA. However, these possibilities are unexamined.

## Methods

This cross-sectional study, conducted from January 1, 2017, to April 30, 2021, implemented an interrupted time series study design^5^ based on electronic health records from Meuhedet, an Israeli nonprofit health maintenance organization (HMO) with national coverage. Israeli legislation dictates that each citizen must join an HMO. HMOs are mandated to accept members regardless of medication, diagnostic, or demographic factors (ie, selection bias would be illegal). The interval before March 1, 2020, was classified as unexposed to the pandemic; the interval starting on that date was classified as exposed.^6^ The primary analysis quantified the association of the COVID-19 pandemic with the monthly SA incidence rate (eAppendix 1 and eAppendix 2 in Supplement 1). Relative risks (RRs) and 95% CIs were estimated from a Poisson regression model. The regression model validity was verified using diagnostic tools and sensitivity analyses (eAppendix 3 in Supplement 1). Two scenarios were forecast for 12 months after the third wave to project SA rates and their associated 95% prediction intervals (PIs). This report followed the STROBE reporting guideline. The Meuhedet-associated Helsinki institutional review board granted ethical approval to conduct this study with a waiver of written informed consent because the data were deidentified.

## Results

**Figure 1.**
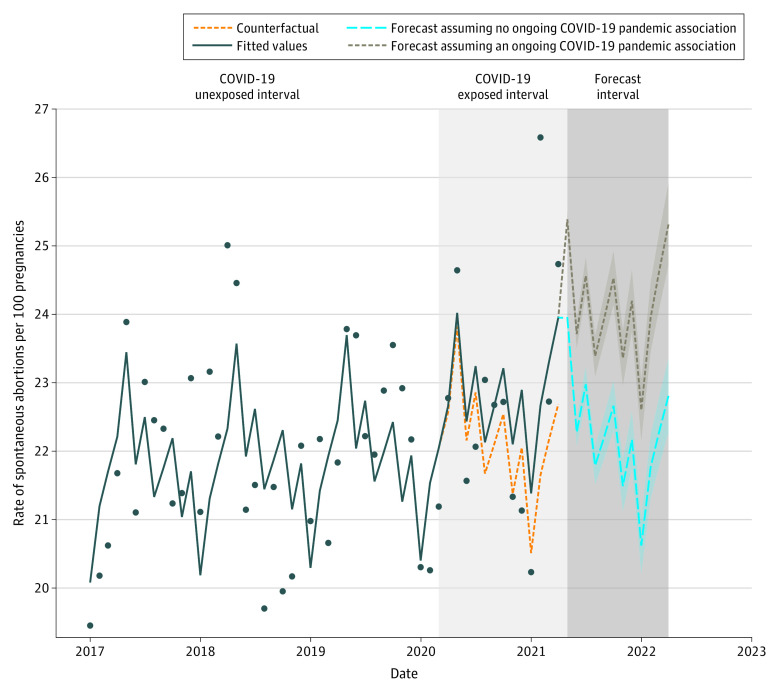
Spontaneous Abortion (SA) Incident Rates: Before, During, and Forecasts After COVID-19 Poisson interrupted time series–fitted values; the model-based counterfactual values, assuming that COVID-19 had not occurred; and 2 forecast scenarios together with the corresponding 95% prediction intervals: assuming no ongoing association of the COVID-19 pandemic with SA rates and assuming an ongoing association of the COVID-19 pandemic with SA rates. Lockdown intervals: March 13 to April 30, 2020 (first lockdown), September 18 to October 17, 2020 (second lockdown), and December 27, 2020, to February 7, 2021 (third lockdown). Dots indicate the observed monthly SA rate.

**Figure 2.**
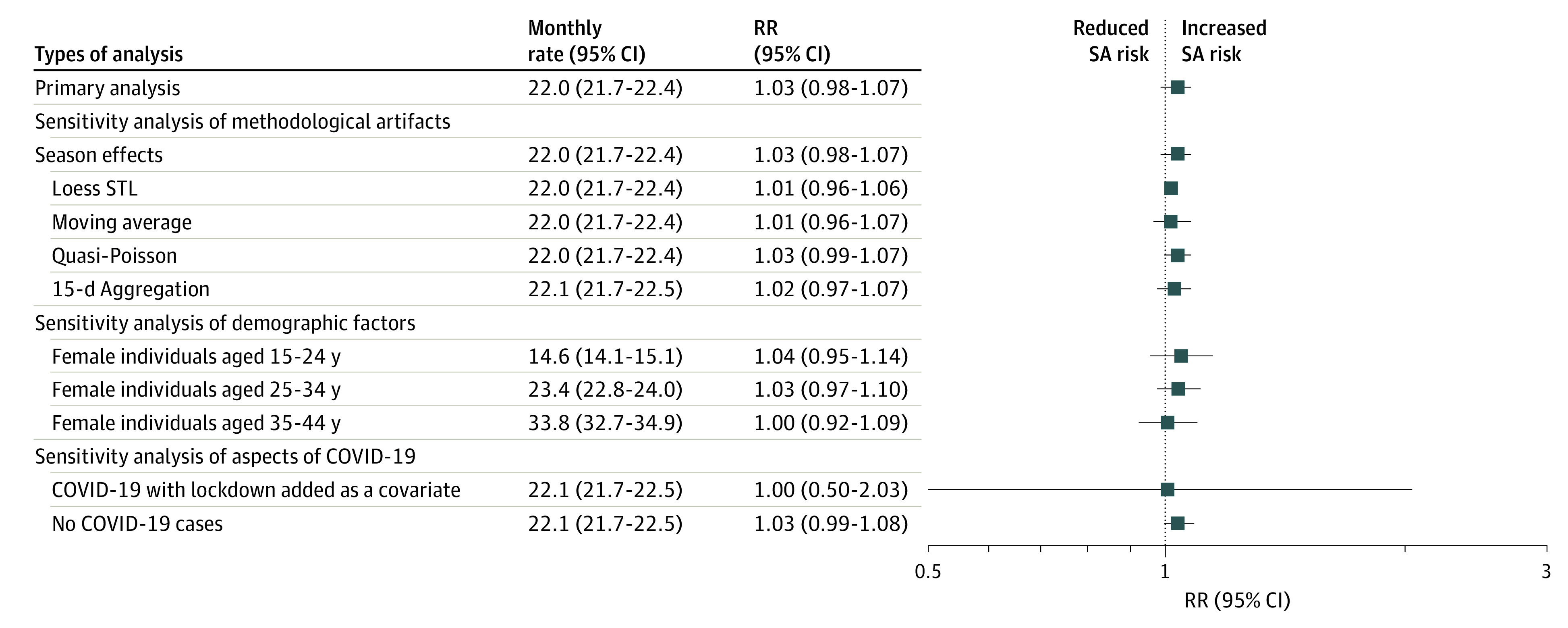
Adjusted Relative Risk (RR) of the Monthly Incident Rate of Spontaneous Abortion (SA), Comparing the Fitted Values With the Model-Based Counterfactual Values For each exposure, the reference group was the model-based counterfactual values, assuming that COVID-19 had not occurred. STL indicates seasonal and trend decomposition using Loess.

The study cohort comprised pregnancies among 252 858 women aged 15 to 44 years, followed up from January 1, 2017, to April 30, 2021, with an estimated 59 142 pregnancies during the study period. During the study, 13 027 incident SAs were observed (rate per 100 pregnancies, 22.0; 95% CI, 21.7-22.4). In the primary analysis, compared with the counterfactual (the exposed interval assuming COVID-19 had not occurred), pandemic exposure had a null association with the monthly SA incident rate (RR, 1.03; 95% CI, 0.98-1.07) (Figure 1). The results remained null across 10 sensitivity analyses testing the robustness of the primary analysis (Figure 2). Forecasts of SA rates per 100 pregnancies 12 months after the end of the third wave were estimated at 22.8 (95% PI, 22.3-23.4), assuming no ongoing association of the pandemic with SA rates, and at 25.3 (95% PI, 24.7-25.9), assuming an ongoing association of the pandemic with SA rates (Figure 1).

## Discussion

The results showed that the RRs of SA rates during 3 pandemic waves did not differ from rates during the 3 prepandemic years. Forecast models suggested that the SA incidence will increase slightly compared with prepandemic levels, even assuming an absence of an association of the pandemic with SA rates. However, pandemic changes may offset our forecasts. This result may reflect ongoing socioeconomic disruptions and COVID-19 anxiety.

This study has some limitations. Due to imprecise timing in our data, we could not examine the association of vaccination or cases of COVID-19 infection with SA rates. Truncation bias seems modest, as data are available until February 2022, which allowed us to estimate all pregnancies until April 2021. Nonetheless, pregnancy is estimated in this study, and so may contain error. We lacked information on repeated pregnancies, socioeconomic status, and other potential confounders, making residual confounding plausible. However, the results, based on interrupted time series analysis, remained null in 10 rigorous sensitivity analyses.

This study suggests that trends in the monthly SA incidence rates in Israel observed before and during the COVID-19 pandemic did not differ. However, forecasting models indicate an anticipated increase, which underscores the need for enhanced public health monitoring for SA prevention.

## Notes

Supplement 1. **eAppendix 1.** Model Details **eAppendix 2.** Definition of Births and Spontaneous Abortions **eAppendix 3.** Sensitivity Analyses
eReferences.

Supplement 2.
Data Sharing Statement
