# Supplementary material for: Rates of Spontaneous Abortion in Israel Before and During the COVID-19 Pandemic
Source: JAMA Netw Open. 2023 Feb 21;6(2):e230233. doi: 10.1001/jamanetworkopen.2023.0233 (PMC9945079; doi:10.1001/jamanetworkopen.2023.0233)
Supplement: Supplement 1. — eAppendix 1. Model Details eAppendix 2. Definition of Births and Spontaneous Abortions eAppendix 3. Sensitivity Analyses eReferences. [file jamanetwopen-e230233-s001.pdf]

Supplementary Online Content

Travis-Lumer Y, Goldberg Y, Kodesh A, et al. Rates of spontaneous abortion in Israel before and during the COVID-19 pandemic. *JAMA Netw Open.* 2023;6(2):e230233. doi:10.1001/jamanetworkopen.2023.0233

eAppendix 1. Model Details
eAppendix 2. Definition of Births and Spontaneous Abortions
eAppendix 3. Sensitivity Analyses
eReferences.

This supplementary material has been provided by the authors to give readers additional information about their work.

© 2023 Travis-Lumer Y et al. *JAMA Network Open.*

eAppendix 1. Model Details

We model the monthly spontaneous abortions count by fitting a Poisson regression model with the following covariates: time (with the month as the underlying time unit), and the COVID-19 exposure interaction with time (where exposure was classified as unexposed or exposed to the COVID-19 pandemic). Note that we added an offset term to the Poisson regression model since it is appropriate to focus on the rate of spontaneous abortion rather than the count. Also, we used Fourier terms in the Poisson model as covariates to model the seasonal component[1]. Seasonal components are periodic oscillations in a time series that occur due to the periodic time effect. For our analysis, we used the three Fourier terms that were statistically significant (P<0.05) in the Poisson model.

Our Poisson regression model can be presented using the following notation; denote by t the time (a sequence of months from 1 to 52, with $t^* = 39$ for March 2020), by Y_t the monthly spontaneous abortions count, by P_t the monthly number of pregnancies, by $X_t = \mathbf{1}_{\{t \geq 39\}}$ the COVID-19 indicator, and by S_t the seasonal component (multivariate Fourier terms). Then our regression model is

$$\log\big(E(Y_t \mid X_t, S_t)\big) = \log(P_t) + \beta_0 + \beta_1 \cdot t + \beta_2 \cdot X_t \cdot (t - t^*) + \gamma^T S_t,$$

where β_0 is the intercept, β_1 is the time trend coefficient, β_2 is the slope change following the intervention, and $\gamma \in \mathbb{R}^3$ is the multivariate coefficient of the Fourier terms.

We then followed recommendations in the literature[2] to estimate the relative risk of the COVID-19 period on spontaneous abortion rates.

© 2023 Travis-Lumer Y et al. *JAMA Network Open.*

eAppendix 2. Definition of Births and Spontaneous Abortions
Live births were recorded in the HMO EHR data. Similarly, all cases of spontaneous abortion (1) required medical attention; (2) were based on ICD codes that were recorded by the treating board-recognized physician; and (3) were under 20 weeks of gestation.

For each month, we observe the number of births and the number of miscarriages that occurred during that month. It is not possible to identify precisely when a specific pregnancy began because in these interrupted time series data we only observe the monthly number of births and the monthly number of spontaneous abortions. However, we can calculate the approximate number of pregnancies beginning each month as follows. We use the following notation. Denote by t the time (a sequence of months from 1 to 52, with t^*=39 for March 2020), by P_t the number of pregnancies beginning in month t, by B_t the number of births at month t, and by SA_t the number of spontaneous abortions at month t. Then the estimated number of pregnancies beginning in month t, P_t, was calculated according to the following formula

$$P_t = B_{t+9} + p_1 \cdot SA_{t+1} + p_2 \cdot SA_{t+2} + p_3 \cdot SA_{t+3} + p_4 \cdot SA_{t+4},$$

where we assume for simplicity, because this is an estimate, that all births occurred precisely nine months after conception (pregnancy start), and where p_i is the probability of a spontaneous abortion at month $t + i$,$1 \leq i \leq 4$, (where the first four months corresponds to the first 20 weeks of pregnancy), and is based on estimates of spontaneous abortion probabilities by week.[3] Accordingly,

$$Y_t = p_1 \cdot SA_{t+1} + p_2 \cdot SA_{t+2} + p_3 \cdot SA_{t+3} + p_4 \cdot SA_{t+4}$$

is the estimated number of spontaneous abortions related to a pregnancy beginning in month t. Note that in the Poisson regression model, Y_t is the response, and P_t is the offset to consider the rate.

© 2023 Travis-Lumer Y et al. *JAMA Network Open.*

eAppendix 3. Sensitivity Analyses

We implemented ten sensitivity analyses to address five methodological artifacts, three demographic factors, and two COVID-19 factors. The first set of five sensitivity analyses of methodological artifacts included three different seasonal adjustments (season effects using simple indicator variables per season, a seasonal-trend decomposition using Loess (STL), and a moving average (MA) seasonal decomposition), a quasi-Poisson model to account for any possible overdispersion, and one additional analysis where we changed the underlying time scale to 15-day intervals to test for aggregation effects. The second set of sensitivity analyses included a separate analysis for each of the three age groups. The third and final set of sensitivity analyses focused on the impact of the severity of social restrictions and COVID-19 infection status. Here, we tested for potential differences in spontaneous abortion incident rates during and not during lockdown periods based on 15-day intervals, and we conducted an additional analysis restricted only to individuals who did not test positive for COVID-19 in each month.

© 2023 Travis-Lumer Y et al. *JAMA Network Open.*

eReferences.

1. Bernal JL, Cummins S, Gasparrini A. Interrupted time series regression for the evaluation of public health interventions: A tutorial. *Int J Epidemiol*.. 2017;46(1):348-355.
2. Travis-Lumer Y, Goldberg Y, Levine SZ. Effect size quantification for interrupted time series analysis: Implementation in R and analysis for Covid-19 research. *Emerg Themes Epidemiol*. 2022;19(1):9.
3. Ammon Avalos L, Galindo C, Li DK. A systematic review to calculate background miscarriage rates using life table analysis. *Birth Defects Res A Clin Mol Teratol*. 2012;94(6):417-423.

© 2023 Travis-Lumer Y et al. *JAMA Network Open.*
