# Supplementary material for: Rates of Spontaneous Abortion in Israel Before and During the COVID-19 Pandemic
Source: JAMA Netw Open. 2023 Feb 21;6(2):e230233. doi: 10.1001/jamanetworkopen.2023.0233 (PMC9945079; doi:10.1001/jamanetworkopen.2023.0233)
Supplement: Supplement 2. — Data Sharing Statement [file jamanetwopen-e230233-s002.pdf]

Data Sharing Statement

Travis-Lumer. Rates of Spontaneous Abortion in Israel Before and During the COVID-19 Pandemic. *JAMA Netw Open*. Published February 21, 2023.
doi:10.1001/jamanetworkopen.2023.0233

Data
Data available: No

Additional Information
Explanation for why data not available: Due to data security and privacy we are unable to make these data that are from electronic healthcare records available.
